# Tumour growth rate predicts overall survival in patients with recurrent WHO grade 4 glioma

**DOI:** 10.1186/s12880-024-01263-y

**Published:** 2024-05-27

**Authors:** Jeffer Hann Wei Pang, Seyed Ehsan Saffari, Guan Rong Lee, Wai-Yung Yu, Choie Cheio Tchoyoson Lim, Kheng Choon Lim, Chia Ching Lee, Wee Yao Koh, Wei Tsau, David Chia, Kevin Lee Min Chua, Chee Kian Tham, Yin Yee Sharon Low, Wai Hoe Ng, Chyi Yeu David Low, Xuling Lin

**Affiliations:** 1 Department of Neurology, National Neuroscience Institute, 11 Jalan Tan Tock Seng, 308433 Singapore, Singapore; 2 Centre of Quantitative Medicine, Duke-NUS Medical School, National University of Singapore, Singapore, Singapore; 3 Department of Neuroradiology, National Neuroscience Institute, Singapore, Singapore; 4 Department of Diagnostic Radiology, Singapore General Hospital, Singapore, Singapore; 5 Division of Radiation Oncology, National University Cancer Institute, Singapore, Singapore; 6 Department of Radiation Oncology, National Cancer Centre Singapore, Singapore, Singapore; 7 Department of Medical Oncology, National Cancer Centre Singapore, Singapore, Singapore; 8 Department of Neurosurgery, National Neuroscience Institute, Singapore, Singapore

**Keywords:** Glioblastoma, Magnetic resonance imaging, Prognostic factor, Tumour volume

## Abstract

**Purpose:**

Accurate prognostication may aid in the selection of patients who will benefit from surgery at recurrent WHO grade 4 glioma. This study aimed to evaluate the role of serial tumour volumetric measurements for prognostication at first tumour recurrence.

**Methods:**

We retrospectively analyzed patients with histologically-diagnosed WHO grade 4 glioma at initial and at first tumour recurrence at a tertiary hospital between May 2000 and September 2018. We performed auto-segmentation using ITK-SNAP software, followed by manual adjustment to measure serial contrast-enhanced T1W (CE-T1W) and T2W lesional volume changes on all MRI images performed between initial resection and repeat surgery.

**Results:**

Thirty patients met inclusion criteria; the median overall survival using Kaplan-Meier analysis from second surgery was 10.5 months. Seventeen (56.7%) patients received treatment post second surgery. Univariate cox regression analysis showed that greater rate of increase in lesional volume on CE-T1W (HR = 2.57; 95% CI [1.18, 5.57]; *p* = 0.02) in the last 2 MRI scans leading up to the second surgery was associated with a higher mortality likelihood. Patients with higher Karnofsky Performance Score (KPS) (HR = 0.97; 95% CI [0.95, 0.99]; *p* = 0.01) and who received further treatment following second surgery (HR = 0.43; 95% CI [0.19, 0.98]; *p* = 0.04) were shown to have a better survival.

**Conclusion:**

Higher rate of CE-T1W lesional growth on the last 2 MRI images prior to surgery at recurrence was associated with increase mortality risk. A larger prospective study is required to determine and validate the threshold to distinguish rapidly progressive tumour with poor prognosis.

## Background

World Health Organization (WHO) grade 4 glioma is the most common malignant primary brain tumour; it is often rapidly progressive and portends poor prognosis [1]. Current standard treatment comprises maximal surgical resection, radiation therapy and chemotherapy with Temozolomide [2]. Despite treatment, almost all WHO grade 4 glioma recur [3].

At recurrence, there is currently no standard of care [1]. Most patients who are fit for and have lesions amendable to surgery will be offered maximal safe resection. While there have been no randomized controlled trials specifically addressing the survival benefit of surgery, gross total resection has been shown to confer survival benefit [4–6]. Nonetheless, there remains no standard preoperative stratification methods to identify patients who will benefit from surgery at recurrence [7]. In our observation, patients with rapidly progressive tumour may have their postoperative residual tumour grow significantly within 3–4 weeks following surgery and before they are considered safe to start systemic therapy and/or radiation therapy. Arguably, it may be prudent to consider chemotherapy and/or radiation therapy instead of surgery for patients with rapidly growing recurrent tumour, especially when gross total resection is unlikely to be achieved [8].

Hence we conducted this study to evaluate the prognostic role of serial tumour volumetric measurement at WHO grade 4 glioma first recurrence. We hypothesized that the greater the rate of tumour volume increase prior to surgery at first tumour recurrence, the more likely the patient will have a worse survival and potentially less likely to benefit from surgery.

## Methods

### Study settings

In this retrospective study, all patients with histologically diagnosed WHO grade 4 glioma at a tertiary hospital between May 2000 and September 2018 and had undergone a surgical resection with histologically-confirmed tumour recurrence were included. All patients had undergone at least two MRI brain scans between the initial surgery and repeat surgery with images available for review. For the purpose of this study, WHO grade 4 glioma includes both isocitrate dehydrogenase (IDH) mutated and IDH wild-type glioblastoma based on the 2016 WHO classification of tumours of the central nervous system [9]. The study was approved by the Institution Review Board (CIRB: 2017/2439).

### MRI and volumetric assessment

All MRI brain scans were performed under National Neuroscience Institute (NNI) Singapore on a 1.5T MRI scanner (Signa HDxt, General Electric Medical Systems, Milwaukee, WI) or a 3T whole body MRI system (Achieva 3.0, Philips Medical Systems, Best, the Netherlands). Imaging protocols comprised axial T1-weighted (T1W) spin-echo (SE) (repetition time [TR]/echo time [TE]/field of view [FOV]; 500–640 ms/10–12 ms/220–240 mm; matrix 1.5T/3.0T; 320 × 224/256 × 200), axial T2-weighted (T2W) fast/turbo SE (TR/TE/FOV; 3,000 ms/83–100 ms/240 mm; matrix 1.5T/3.0T; 384 × 224/436 × 306), coronal fluid- attenuated inversion recovery (FLAIR) (TR/TE/inversion time/FOV; 10,000–11,000 ms/ 125–128/2,200–2,800/240 mm; matrix 1.5T/3.0T; 256 × 192/368 × 186), single shot echoplanar diffusion-weighted imaging in the axial plane (TR/TE/FOV; 3,378–8,000 ms/ 71–73 ms/240–250 mm; diffusion gradient encoding in 3 orthogonal directions; b 5 1,000 s/mm2; matrix 1.5T/3.0T; 128 × 128/124 × 123), axial gradient echo (TR/TE/FOV; 678– 800 ms/15–25 ms/240 mm; matrix 1.5T/3.0T; 256 × 192/256 × 256), and axial and coronal post-gadolinium/contrast-enhanced T1W (CE-T1W) SE (matrix 1.5T/3.0T; axial: 256 × 256/240 × 188; coronal: 320 × 224/264 × 193).

For each CE-T1W and MRI sequence, we used ITK-SNAP, a software application for image segmentation and volume calculation, to perform auto-segmentation followed by manual adjustment of the segmented region-of-interest (ROI) by an internal medicine trainee. The ROIs included hyperintense areas on the T2W images and enhancing components on the CE-T1W images. Non-enhancing areas within CE-T1W lesions which may represent necrosis or cysts, were included in the “tumour volume” during our measurement [10]. On the other hand, blood products and chemical haemostatic agents such as Surgicel (Ethicon, Somerville, NJ) used intraoperatively can be hyperintense in the pre-gadolinium T1W images. For this reason, we excluded lesion that were hyperintense on pre-gadolinium T1W images. However areas within CE-T1W enhancing regions that represented intratumoural haemorrhage were included in the ROIs.

We recorded the calculated lesional volume on CE-T1W and T2W images for each MRI scan and compared the results across serial MRI scans for each patient. All lesional segmentation and volumetric measurements were verified independently by a neuro-oncologist. RadiAnt Dicom viewer version 2020.1 (Trial version) was used for the observation of MRI images when needed.

### Data analysis

Patients’ demographics, treatments and clinical features were reported as frequency and percent for categorical variables, and mean, median, minimum and maximum for continuous variables. Survival analysis was performed to investigate the association of demographics and clinical parameters with overall survival outcome. Un-adjusted hazard ratio and 95% confidence intervals were calculated using cox regression analysis. Overall survival is defined as date of surgery at first tumour recurrence to death. Median overall survival was calculated using Kaplan-Meier analysis.

Rate of CE-T1W and T2W lesional volume change were calculated (i) between the first scan post-initial surgery and the last scan prior to surgery at tumour recurrence, and (ii) between the last 2 scans prior to surgery at recurrence. Rate of lesional volume change is calculated as volume change over time between the respective scans. Univariate cox regression models were then conducted to investigate the association between the rate of lesional change on serial volumetric measurement with overall survival from date of surgery at recurrence; hazard ratios and 95% confidence intervals were reported.

When performing cox regression analysis, the proportional hazard ratio assumption was assessed via Kolmogorov-type supremum test using simulated patterns. In addition, we calculated the proportion of patients with good functional score, defined as KPS >/= 70 and those with poor functional score (KPS < 70) who did not receive further postoperative treatment. Chi-square was conducted to compare the proportions between the groups.

Statistical significance was set at *p* < 0.05. Data analysis was performed in SAS software version 9.4 for Windows (Cary, NC: SAS Institute Inc.).

## Results

### Patient characteristics

A total of 30 patients met inclusion criteria. There were 18 men and 12 women. The median age was 53.5 (range 17–75) years and the median KPS was 80 (range 30–90) at initial diagnosis; 3 patients do not have KPS evaluable. All tumours were located supratentorial and the majority (60%) underwent gross total resection. Postoperatively, all 27 patients with evaluable KPS had stable or improved KPS (range 70–90) compared to their preoperative KPS. Among all 30 patients, 25 received postoperative chemo-radiation, 1 had radiation therapy only and 4 did not undergo any subsequent treatment (2 patients were lost to follow-up shortly after surgery but returned at tumour recurrence, and 2 patients declined treatment).

The median duration from initial diagnosis to surgery at tumour recurrence was 13 (range 2 to 90) months. Among the 27 patients with evaluable KPS, median KPS prior to surgery at tumour recurrence was 80 (range 30–90). Following surgery at tumour recurrence, 6 patients developed postoperative complications of right middle cerebral artery infarct (*n* = 4), infection (*n* = 1) and intraventricular hemorrhage associated with hydrocephalus (*n* = 1) and had stable or worse KPS compared to their preoperative KPS; the remaining 21 patients with evaluable KPS had stable to improved KPS (median 90; range 40–90).

Five patients had Carmustine Wafer implanted into the surgical cavity during surgical resection at tumour recurrence and did not receive further systemic therapy; 10 patients received immediate postoperative systemic therapy (1 Bevacizumab, 4 Lomustine, 4 Temozolomide, 1 unknown) and 3 patients had radiation therapy; 9 patients did not receive immediate postoperative treatment and 3 patients had limited records to verify their treatment regimen (Table 1). Among the 9 patients who did not receive treatment, 4 had poor KPS (KPS < 70), 3 declined treatments, 1 had rapidly progressive tumour and deceased after 4 months, and 1 was readmitted for seizures and was subsequently lost to follow-up. At further tumour recurrences, 2 of the 3 patients who had declined immediate postoperative treatment received further treatment including repeat surgeries, Bevacizumab and radiation therapy.

**Table 1:** Patients’ clinical data and radiological volumetric measurement (*N* = 30)

Clinical Data		N	%
Gender	Female	12	40.0
	Male	18	60.0
Ethnicity	Chinese	21	70.0
	Malay	4	13.3
	Indian	3	10.0
	Eurasian	1	3.3
	Sikh	1	3.3
Extent of initial surgery	Gross total	18	60.0
	Subtotal	10	33.3
	Biopsy	2	6.7
IDH	Wild	12	40.0
	Mutated	2	6.7
	Unknown	16	53.3
KPS prior to initial surgery	>/=70	24	80.0
	< 70	3	10.0
	Unknown	3	10.0
KPS post initial surgery	>/=70	27	90.0
	< 70	0	0.0
	Unknown	3	10.0
Treatment after initial surgery	Chemoradiation	25	83.3
	Radiation therapy only	1	3.3
	Chemotherapy only	0	0.0
	None	4	13.3
KPS prior to surgery at tumour recurrence	>/=70	22	73.3
	< 70	5	16.7
	Unknown	3	10.0
KPS prior to surgery at tumour recurrence	>/=70	20	66.7
	< 70	7	23.3
	Unknown	3	10.0
Carmustine wafer implanted during surgery at tumour recurrence	Yes	5	16.7
	No	25	83.3
Treatment after surgery at tumour recurrence	Chemoradiation	0	0.0
	Radiation therapy only	3	10.0
	Chemotherapy only	10	33.3
	None (including those with carmustine wafer)	14	46.6
	Unknown	3	10.0
Mortality at study analysis	No	25	83.3
	Yes	5	16.7
**Radiological Volumetric Measurement**			
T1W-post gadolinium volume at last MRI prior to surgery at recurrence, mm^3^	Mean	45,361	
	Median	30,585	
T2W volume at last MRI prior to surgery at recurrence, mm^3^	Mean	161,976	
	Median	149,800	
Rate of T1W-post gadolinium volume change between first post initial surgery scan and last scan prior to surgery at recurrence, mm^3^/day	Mean	195	
	Median	56.13	
Rate of T2W volume change between first post initial surgery scan and last scan prior to surgery at recurrence, mm^3^/day	Mean	537	
	Median	179	
Rate of T1W-post gadolinium volume change between the last two MRI (prior to surgery at recurrence), mm^3^/day	Mean	275	
	Median	150	
Rate of T2W volume change between the last two MRI (prior to surgery at recurrence), mm^3^/day	Mean	1812	
	Median	798	

Abbreviations CE-T1W, contrast-enhanced T1-weighted; IDH, isocitrate dehydrogenase; MGMT, O^6^-methylguanine-DNA methyltransferase; T2W, T2-weighted

At study completion, 25 of 30 patients had deceased; the median overall survival was 10.5 months.

### Volumetric analysis

For the whole cohort, the mean and median T2W lesional volume on the last scan prior to surgery at recurrence were 161,976 mm^3^ and 149,800 mm^3^ respectively, and the mean and median CE-T1W lesional volume were 45,361 mm^3^ and 30,585 mm^3^ respectively. Between the first post-initial surgery scan and the last scan prior to surgery at recurrence, the median rate of T2W lesional volume change was 179 mm^3^/day respectively, and the median rate of CE-T1W change was 56 mm^3^/day. On evaluation of the last 2 MRI scans prior to the surgery at recurrence, the median rate of T2W lesional volume change was 798 mm^3^/day and the median rate of CE-T1W lesional volume change was 150 mm^3^/day (Table 1).

On univariate cox regression analysis, we found that increase rate of lesional volume change on CE-T1W (HR = 2.57; 95% CI [1.18, 5.57]; *p* = 0.02) but not T2W (HR = 0.84; 95% CI [0.62, 1.12]; *p* = 0.23) sequences, in the last 2 MRI scans leading up to the surgery at tumour recurrence, was significantly associated with worse survival. In addition, patients with higher KPS before surgery at recurrence (HR = 0.97; 95% CI [0.95, 0.99]; *p* = 0.01) and who received further treatment following second surgery (HR = 0.43; 95% CI [0.19, 0.98]; *p* = 0.04) were shown to have better survival (Table 2).

**Table 2:** Association of clinical parameters and rate of lesional volume change with overall survival at recurrence: univariate cox regression analysis

Variable	Event vs. Reference Level / Unit	Un-Adjusted Hazard Ratio (95% CI)	P value
Age at initial surgery	Year	1.039 (0.999, 1.081)	0.0588
Age at surgery at recurrence	Year	1.039 (0.999, 1.081)	0.0586
Gender	Female vs. Male	1.414 (0.631, 3.169)	0.4005
Treatment post-surgery at recurrence	Yes vs. No	0.432 (0.190, 0.980)	0.0446
T1W-post gadolinium volume*	mm^3^	1.058 (0.735, 1.524)	0.7598
T2W volume*	mm^3^	1.377 (0.866, 2.190)	0.1758
Rate of T1W-post gadolinium volume change between first post initial surgery scan and last scan prior to surgery at recurrence*	mm^3^/day	1.199 (0.694, 2.072)	0.5158
Rate of T2W volume change between first post initial surgery scan and last scan prior to surgery at recurrence*	mm^3^/day	1.208 (0.758, 1.927)	0.4266
Rate of T1W-post gadolinium volume change between the last two MRI (prior to surgery at recurrence)*	mm^3^/day	2.565 (1.182, 5.567)	0.0172
Rate of T2W volume change between the last two MRI (prior to surgery at recurrence)*	mm^3^/day	0.835 (0.623, 1.118)	0.2259

* Hazard ratio is calculated for one standard deviation change

Abbreviations CE-T1W, contrast-enhanced T1-weighted; T2W, T2-weighted

Interestingly, there was also no significant difference in the proportion of patients who received further postoperative treatment among patients with good functional score (KPS >/= 70) and with poor functional score (KPS < 70) (*p* = 0.12) (Table 3).

**Table 3:** Correlation of functional status with postoperative treatment at tumour recurrence

Variable	Category	Received postoperative treatment		
		No	Yes	p value^*^
KPS after 2nd surgery				0.122
	< 70	7 (70%)	3 (30%)	
	>/=70	7 (35%)	13 (65%)	

^*^ Chi-square test

Abbreviation KPS, Karnofsky Performance Score

## Discussion

Consistent with our hypothesis, patients had poorer prognosis when their tumours grew more rapidly prior to their surgery at recurrence. Specifically, patients were 2.6 times more likely to have a poor prognosis if the contrast-enhancing component of their tumours were growing rapidly. This finding support the prognostic role of serial CE-T1W volumetric measurements at first recurrence of WHO grade 4 glioma.

WHO grade 4 glioma typically shows gadolinium enhancement on T1W images. The enhancing areas reflect parenchymal areas where gadolinium-based contrast agent has leaked out of the blood-brain barrier into the interstitial tissues and is correlated with higher tumour grade [11]. In contrast, the non-enhancing T2W hyperintense peripheral zones are usually vasogenic oedema with irregular number of neoplastic cells. In pathology studies, as high as 90% of tumour recurrences were reported to have taken place within this T2W hyperintense region [12]. However, T2W hyperintense areas may also be due to post-surgical and post-radiation inflammatory changes [11]. Hence we measured T1W-gadolinium enhancing regions and T2W regions separately in our study. These differences in the radiological implications may account for the lack of correlation of T2W lesional volume changes with patient survival, as compared to that for CE-T1W lesional volume changes.

There is currently no standard treatment for recurrent WHO grade 4 glioma and management is typically guided by a multi-disciplinary team comprising neurosurgeons, neurooncologists, radiation oncologists, radiologists and pathologists. While there have been no randomized controlled trials specifically addressing the survival benefit of surgery, a subgroup analysis of the prospective DIRECTOR trial indicated that gross total resection may improve survival and quality of life [13]. A retrospective matched cohort study evaluating the role of repeat surgery and salvage therapies for recurrence also demonstrated survival benefit primarily limited to gross total resection [14]. In addition, prognosis at tumour recurrence after surgery varies among patients. Older age [5, 15], poor performance status [5, 16, 17], large preoperative tumour size [17, 18] and short time interval between initial and surgery at first recurrence [15, 19] have been correlated with worse prognosis in some studies but not in others [20]. Using KPS, tumour involvement of prespecified eloquent regions and tumour volume, J Park et al. devised and validated a pre-operative additive scale which was able to distinguish patients with good, intermediate, and poor postoperative survival [17]. A later study developed and validated a different 3-tier additive scale which incorporated only two variables: KPS and ependymal involvement. However both studies did not evaluate the prognostic role of serial tumour growth. Our study showed that lower KPS and omission of postoperative treatment but not age correlated with worse survival. Similar to the study by Quick et al. [20], we did not find the absolute preoperative tumour size or time between initial and surgery at first recurrence to be prognostic. Instead we demonstrate that the rate of change, a function of tumour size over time, correlated with survival outcome. Hence, our results showing a significant difference in prognosis between the rapidly growing and the slow growing tumour suggest that serial volumetric assessment of CE-T1W enhancing regions may be a more accurate, innovative method to prognosticate and stratify patients for invasive surgery.

There has been growing interest in the growth rate of grade 4 gliomas. In fact, higher growth rates pre-treatment have been shown to correlate with worse survival time [21, 22]. Furthermore, IDH1 and TERT mutations, 1p19q codeletion and MGMT methylation status were shown to be independently associated with tumour growth, providing molecular basis for the worse outcome [23]. To our knowledge, our study is the first to demonstrate the prognostic implication of tumour growth rate at tumour recurrence.

Interestingly, patients’ functional status using KPS after surgery at recurrence did not correlate significantly with further postoperative treatment. This suggests that rate of tumour growth prior to surgery at tumour recurrence may be more predictive of patients’ eligibility for postoperative treatment than postoperative functional status.

Our study has several limitations. First, this is a retrospective analysis with its inherent bias. Second, our cohort size is small and limits our ability to investigate the impact of correcting confounders that may influence the effect of rate of CE-T1W volume change on patient survival. Third, we included patients with WHO grade 4 glioma IDH-wild type (glioblastoma), IDH-mutated and unknown IDH mutational status. Despite this heterogeneous group, we were able to demonstrate a significant prognostic value of serial CE-T1W volumetric assessment of enhancing tumours at first tumour recurrence. We performed further analysis and found no significant difference in survival at recurrence between patients with IDH-wild type and those with IDH-mutated or unknown IDH mutational status (*p* = 0.7). Fourth, there could be variability in the manual adjustment of ROIs. We sought to reduce variability by having a single operator, an internal medicine trainee, adjust the ROIs, supervised by a neuro-oncologist with 7 years’ experience. We deliberately chose the use of an online software application for image segmentation and volume calculation by a non-radiology specialist operator to ensure that our study methodology could be easily adopted and implemented in routine clinical practice in future.

In conclusion, our study demonstrated the prognostic value of rate of CE-T1W volume change at last 2 MRIs prior to surgery at first WHO grade 4 glioma recurrence. A larger prospective study will be required to validate and determine the threshold of rate of CE-T1W volume change that can distinguish rapidly progressive tumour with poor prognosis and slow growing tumour with better prognosis, and prospectively test its use, together with other known prognostic markers, for stratifying patients for invasive surgery at tumour recurrence.

## Data Availability

The datasets used and/or analysed during the current study are available from the corresponding author on reasonable request.
